# Dietary Conjugated Linoleic Acid Modulates the Hepatic Circadian Clock Program *via* PPARα/REV-ERBα-Mediated Chromatin Modification in Mice

**DOI:** 10.3389/fnut.2021.711398

**Published:** 2021-10-15

**Authors:** Hao-Yu Liu, Haotian Gu, Yanwei Li, Ping Hu, Yatian Yang, Kaiqi Li, Hao Li, Kexin Zhang, Bo Zhou, Huaxing Wu, Wenbin Bao, Demin Cai

**Affiliations:** ^1^Laboratory of Animal Physiology and Molecular Nutrition, College of Animal Science and Technology, Yangzhou University, Yangzhou, China; ^2^Department of Biochemistry and Molecular Medicine, UC Davis School of Medicine, Sacramento, CA, United States; ^3^Institute of Digestive Disease, Zhengzhou University, Zhengzhou, China; ^4^Baijiu Science and Research Center, Sichuan Swellfun Co., Ltd., Chengdu, China

**Keywords:** circadian clock, metabolic physiology, nuclear receptor, PPARα, REV-ERBα

## Abstract

**Scope:** Disruptions of circadian rhythm cause metabolic disorders and are closely related to dietary factors. In this study, we investigated the interplays between the dietary conjugated linoleic acid (CLA)-induced hepatic steatosis and the circadian clock regulation, in association with lipid homeostasis.

**Methods and Results:** Exposure of mice to 1.5% dietary CLA for 28 days caused insulin resistance, enlarged livers, caused hepatic steatosis, and increased triglyceride levels. Transcriptional profiling showed that hepatic circadian clock genes were significantly downregulated with increased expression of the negative transcription factor, REV-ERBα. We uncovered that the nuclear receptor (NR) PPARα, as a major target of dietary CLA, drives REV-ERBα expression *via* its binding to key genes of the circadian clock, including *Cry1* and *Clock*, and the recruitment of histone marks and cofactors. The PPARα or REV-ERBα inhibition blocked the physical connection of this NR pair, reduced the cobinding of PPARα and REV-ERBα to the genomic DNA response element, and abolished histone modifications in the CLA-hepatocytes. In addition, we demonstrated that CLA promotes PPARα driving REV-ERBα transcriptional activity by directly binding to the PPAR response element (PPRE) at the *Nr1d1* gene.

**Conclusions:** Our results add a layer to the understanding of the peripheral clock feedback loop, which involves the PPARα-REV-ERBα, and provide guidance for nutrients optimization in circadian physiology.

## Introduction

The circadian clock is a time program created endogenously in all organisms. It is controlled by genetically encoded molecular components, which generate cyclic changes in activities during a period of about a day (1, 2). A wide array of metabolic processes displays circadian rhythms, which are linked with sleep-wake cycles, feeding behavior, and nutrient availability (3). Disruptions of the circadian clock may cause metabolic diseases in humans and animals. For example, the incidence rates of obesity and cancer are high in long-shift workers (4, 5). The Clock mutant mice are described to develop a metabolic syndrome of hyperlipidemia, hepatic steatosis, and hypoinsulinemia (6).

At the heart of the molecular circadian clock lies a transcription-translation feedback loop (TTFL), containing the key proteins CLOCK, BMAL1 (*Arntl1*), PERIOD (PER homologs 1–3), and cryptochrome (CRY homologs 1 and 2) (3, 7). The CLOCK-BMAL1 heterodimer is responsible for activating the transcription of target genes in their promoter and/or enhancer regions, including the genes encoding PER and CRY (8, 9), whereas the PER and CRY act as repressors to antagonize the CLOCK-BMAL1 through posttranslational inhibition (10–12). Alternatively, REV-ERBα and REV-ERBβ mediate transcriptional repression of the clock genes in the negative feedback loop (13, 14). The nuclear receptors (NRs), REV-ERBs are ligand-regulated transcription factors (15) that link circadian rhythms with metabolism. REV-ERBα has been shown to regulate the expression of *Arntl1* and affects the lipid signaling protein, apolipoprotein CIII in the liver cells (10). Dual depletion of *REV-ERB*α*/*β function by the creation of double-knockout mice (DKOs) results in multiple phenotypes including altered circadian wheel-running behavior and lipid dysregulation (13). The severity of the circadian disruption was comparable to that was seen in *Bmal1*^−/−^ mice (16). Bmal1 deficiency also reprogram lipid metabolism in the livers of mice (17). Finally, the TTFL is also involved in chromatin modifications of the central circadian oscillators in mammals and plants (18). Dynamic histone modifications can change the chromatin structure of genes, and affect the accessibility of clock regulators and/or RNA polymerase II (Pol-II); thus, promote the rhythmic expression of target genes (19, 20). However, it is still unclear which diverse manners of the molecular clocks are functionally integrated and exhibit distinct tissue-specific transcription regulations (3).

Reciprocally, changes in metabolism can regulate circadian physiology (21). The circadian clock responds to zeitgebers (the so-called time givers) differently (22), depending on both the strength of the stimuli and the circadian phase during which the zeitgebers are applied. In particular, the nutritional challenges, such as high-fat feeding can serve as a strong zeitgeber for the liver, which results in clock reprogramming in mice (21). On administering a high protein/low carbohydrate diet to mice, the expression of BMAL1 and Cry1 were seen to be increased in both the livers and the kidneys (23). Interestingly, it is reported that conjugated linoleic acid (CLA) addition contributes to the alteration of the circadian clock gene expressions in Caco-2 cells *in vitro*, in association with the increased expression of miR-107, while the *in vivo* effects are unknown (24). The CLA is a natural polyunsaturated fatty acid (FA), derived from meat and dairy products (25). The consumption of CLA is related to the reduction of body weight in humans, thereby becomes popular (26). In the western world, the estimated CLA intake ranges from 90 mg per day in the US to 1,500 mg per day in Australia. However, the suggested beneficial intake of CLA is about 3–6 g per day (26, 27). Notably, studies using experimental animals often employ higher doses of CLA than in humans, and produce a considerable variation, even severe side effects including lipoatrophy in rodents (25, 26). Therefore, from the perspective of health science, it is important to better understand the action mechanism of the CLA.

In this study, we hypothesized that a high dose of CLA supplementation may cause changes in the circadian clock program and the associated lipid metabolism in mice. We found that dietary CLA induced hepatic steatosis and insulin resistance in mice during a period of 28 days, and downregulated the clock gene expressions in the liver. By targeting the associated NRs with small-molecule drugs, we aimed to investigate the nature of interactions between the CLA and the molecular clock in animal physiology.

## Materials and Methods

### Animals and Experimental Design

All experiments involving animals were reviewed and approved by the Animal Care and Use Committee of Yangzhou University (YZUDWSY 2017-09-06). Wild-type male Kunming mice at 5 weeks old were purchased from the Jiangsu Laboratory Animals Science Center, housed at 22°C with 50% of humidity on a 12/12-h light/dark cycle and were allowed free access to water and food. Mice were fed a normal chow diet for 1 week for acclimation and were randomized into two groups (*n* = 6): the control group/vehicle received a chow diet with 1.5% (w/w) linoleic acid supplementation, whereas the CLA group received a chow diet with CLA supplementation (1.5% w/w). For *in vivo* studies, the CLA used was a mixture of an equal amount of c9t11 and t10c12 (26). The experiment lasted for 28 days, during which the body weight and food intake were recorded. At the end of the experiment, the animals were euthanized with anesthesia followed by pelltobarbitalum natricum (GENIA Biotech, Beijing, China) (80 mg kg^−1^) treatment. The sampling time was chosen at zeitgeber time (ZT) 8 (8 h after lights are turned on) according to previous descriptions (28). Whole blood was collected *via* cardiac draw with a capillary tube (100 μl, coated with K3 EDTA, Sarstedt, Nümbrecht, Germany), and plasma was prepared by centrifugation. Together with the liver tissues, the samples were taken and were snap-frozen in liquid nitrogen, thereafter stored at −80°C until further analysis.

For the circadian rhythm measurement, a total of 72 mice in two groups were fed as described above for 28 days. The livers were collected from control (*n* = 6) or CLA (*n* = 6) mice at ZT0, ZT4, ZT8, ZT12, ZT16, and ZT20 for 24 h. The liver tissues were snap-frozen in liquid nitrogen for further analysis.

### Lipid, Blood Glucose, Insulin Level Analyses, and Insulin Tolerance Test/Glucose Tolerance Test

Liver triglyceride levels were determined on a Roche Integra 400 Plus analyzer (Roche Diagnostics, Basel, Switzerland). Plasma insulin and glucose levels were measured using a 125I-labeled insulin radioimmunoassay kit (Beijing North Institute of Biological Technology, Beijing, China) and OneTouch Ultra Easy (LifeScan, Shanghai, China) following the instructions of the manufacturers, respectively. For insulin tolerance test/glucose tolerance test (ITT/GTT), the mice had been fasted for 6 h and then gave an intraperitoneal (*i.p.)* injection of insulin (Novolin R; Novo Nordisk, Bagsvaerd, Denmark) or glucose at a dose of 1 unit kg^−1^ or 2 g kg^−1^ body weight. The measurement of blood glucose was conducted by tail bleeding at 0, 30, 60, 90, and 120 min, using the One Touch Ultra Easy.

### Histology and Oil Red O Staining

Five μm thick sections from the liver tissues were stained with hematoxylin and eosin (H&E) and were visualized using a light microscope (Leica DFC 420C; Leica Microsystems, Wetzlar, Germany) at x20 magnification. In parallel, sections were fixed with 10% formalin. After fixation, 60% of isopropanol was added for 30 s and was subsequently removed. Oil Red O working solution (Sunshinebio, Nanjing, China) was then applied for 1 h. After the removal of the Oil Red O working solution, 100% of isopropanol was added to extract Oil Red O and was finally visualized. Duplicate sections of each mouse were used for histological analysis.

### Primary Murine Hepatocytes Isolation and Cell Culture

Hepatocytes were isolated from the control and the CLA-treated mice by liver perfusion using a two-step method with collagenase as described previously (29). Peripheral blood and cells were flushed from the liver in Hank's balanced salt solution followed by perfusion using the collagenase digestion solution. Thereafter, the liver was dissected and mechanically dissociated. The acquired cell suspension was filtered through a 100 μm-cell mesh followed by centrifugation at 50 x *g* for 5 min at 4°C to obtain hepatocytes and were maintained in OptiCulture hepatocyte media (Sekisui XenoTech LLC, KS, USA).

### Real-Time Quantitative Reverse Transcription PCR and Western Blotting Analysis

Total RNA was isolated from mice livers or hepatocytes. The complementary DNA (cDNA) was prepared, amplified, and measured in the presence of SYBR Green as previously described (30). The fluorescent values were collected and a melting curve analysis was performed. *Gapdh* was used as the internal reference to normalize the relative level of each transcript. The primers used are shown in Supplementary Table 1. Hepatocytes were analyzed by immunoblotting with PPARα (Millipore, MA, USA; MAB3890), REV-ERBα (cell signaling, #13418), and Bmal1 (cell signaling, #14020).

### RNA-Sequencing Analysis

The liver tissue RNA was prepared for RNA-sequencing (RNA-seq) analysis using Illumina Tru-Seq RNA Sample Prep Kit (Illumina Inc., CA, USA), according to the instructions of the manufacturer. Libraries were constructed and validated with an Agilent Bioanalyzer (Agilent Technologies, Palo Alto, CA, USA). Sequencing was performed on an Illumina HiSeq 2000 sequencer at BGI Tech (Hong Kong, China). The FASTQ-formatted sequence data were obtained and analyzed using a standard BWA-Bowtie-Cufflinks workflow. Sequence reads were mapped to mm9 assembly with BWA and Bowtie software. The Cufflinks package was used for transcripts assembly, quantification of normalized gene and isoform expression, and the analysis of different expressions. Gene set enrichment analysis (GSEA v.3.0) was applied to rank the genes based on the shrunken limma log2 fold-changes. The GSEA tool was used in a “pre-ranked” model with default parameters.

### Small Interfering RNA Transfection

Small interfering RNAs (siRNAs) for PPARα gene knockdown were purchased from Dharmacon (J-040740-09-0005, J-040740-10-0005). The siRNAs for REV-ERBα gene knockdown were purchased from Dharmacon, CO, USA (J-051721-05-0002, J-051721-06-0002). Transfections were performed with OptiMEM (Invitrogen, MA, USA) and Dharmafectin#1 (Dharmacon, CO, USA) following the instructions of the manufacturers.

### Chromatin Immunoprecipitation Quantitative Real-Time PCR

Mice livers or primary murine hepatocytes were subject to crosslinking in 1% of formaldehyde for 5 min followed by quenching with glycine for 5 min on ice. Cells were pelleted by centrifugation and resuspended in a lysis buffer (50 mM HEPES pH 8.0, 140 mM NaCl, 1 mM EDTA, 10% glycerol, 0.5% NP40, 0.25% Triton X100). The pellets were then resuspended in a washing buffer (10 mM Tris pH 8.0, 1 mM EDTA, 0.5 mM EGTA, 200 mM NaCl), washed and resuspended in a shearing buffer (0.1% SDS, 1 mM EDTA, pH 8, 10 mM Tris HCl, pH 8) before sonication following the instruction of the manufacturer. Chromatin fragments were precipitated using specific antibodies and protein G beads, washed, and treated with proteinase K and RNase A. Purified chromatin immunoprecipitation (ChIP) DNA was then used for chromatin immunoprecipitation quantitative real-time PCR (ChIP-qPCR) analysis. The antibodies used for the ChIP-qPCR assay include PPARα (Millipore, MA, USA; MAB3890); RNAPII (Santa Cruz Biotechnology, TX, USA; sc-899); RNAPII-S2P (Active Motif, CA, USA; #61083); H3K27ac (Abcam, Cambridge, UK; ab4729); H3K4me1(Abcam, Cambridge, UK; ab8895); IgG (Santa Cruz Biotechnology, TX, USA; sc-2027), and REV-ERBα (Proteintech, IL, USA; 14506-1-AP). ChIPs were performed with each experimental point in triplicate, and each experiment was repeated three times. The primers are shown in Supplementary Table 1.

### ChIP-Seq Data Analysis

ChIP-seq Fastq files (GSE61817, GSE67973) were processed by the pipeline of AQUAS Transcription Factor (https://github.com/kundajelab/chipseq_pipeline) as described (31). Briefly, sequencing tags were mapped against mm9 using BWA 0.7.15. (32). Uniquely mapped tags after filtering and deduping were used for peak calling by the model-based analysis for ChIP-Seq (MACS; 2.1.0) to identify the regions of enrichment over the background. Normalized genome-wide signal-coverage tracks from raw-read alignment files were built by MACS2, UCSC tool (bedGraphToBigWig/bedClip; http://hgdownload.cse.ucsc.edu/admin/exe/linux.x86_64/), and bedTools (https://github.com/arq5x/bedtools2). Visualization of ChIP-seq signal at enriched genomic regions (avgprofile and heatmap) was achieved by using deepTools (https://deeptools.readthedocs.io/en/develop/index.html).

### ChIP-Re-ChIP Assays

Chromatin immunoprecipitation was performed as described previously (33) with the following modifications. The crude chromatin solutions of the CLA-treated hepatocytes were first cleared with protein A beads (Invitrogen, MA, USA) that had been precoated with preimmune serum for 2 h at 4°C. Then, the precleared chromatin solutions were incubated at 4°C overnight with the other antibodies listed in the Supplementary Material, prior to precipitation with protein A beads that had been preblocked with bovine serum albumin and sonicated salmon sperm DNA. For re-ChIP, the immunoprecipitated complexes from the first ChIP were eluted with 20 mM dithiothreitol at 37°C for 30 min with brief vortexing, diluted with the ChIP buffer for 50 times, and cleared by centrifugation, before they were incubated with antibodies for the secondary ChIP overnight at 4°C. The immunoprecipitated DNA was analyzed by real-time PCR. Enrichment of genomic DNA was presented as the percent recovery relative to the input. The primers are listed in Supplementary Table 1.

### Reporter Constructs and Reporter-Gene Assay

Transient transfection and reporter-gene assays were performed according to our previous study (30). For reporter-gene assays, the oligonucleotides encompassing four copies of PPAR element (PPRE) motifs found in the murine *Nr1d1* promoter and enhancer were inserted into the tk-luciferase reporter vector. The mutant form, tk- *Nr1d1*-promoter-mu contains sequences mutated from TCAGGGTGAGGTCA to TCAGGGTGCAAGGA. The mutant form, tk-*Nr1d1*-enhancer-mu contains sequences mutated from GGATTAACTAGGTCA to GGATTAACTCAAGGA. Cells (HepG2) were cotransfected with CMX-mPPARα with wild-type or mutant forms of *Nr1d1* promoter and enhancer reporter constructs. The Renilla plasmid was co-transfected for normalization. After 12 h of incubation, the cells were treated with vehicle or CLA or cotreated with compounds as indicated for another 24 h. The luciferase was then analyzed with a Dual-Luciferase Assay system (Promega, WI, USA) on a luminometer according to the instructions of the manufacturer. All transfections were performed in sextuplicate, and each experiment was repeated at least three times.

### Statistical Analysis

Statistical analysis was performed with GraphPad Prism software 8.0. The data are presented as mean ± SD/SEM from at least three independent experiments. Statistical analysis was conducted using two-tailed Student's *t*-tests or ANOVA with Tukey's *post hoc* test to compare the means. The value of *p* < 0.05 was considered significant.

## Results

### Dietary Conjugated Linoleic Acid Induces Nonalcoholic Fatty Liver Disease in Mice

**Figure 1:**
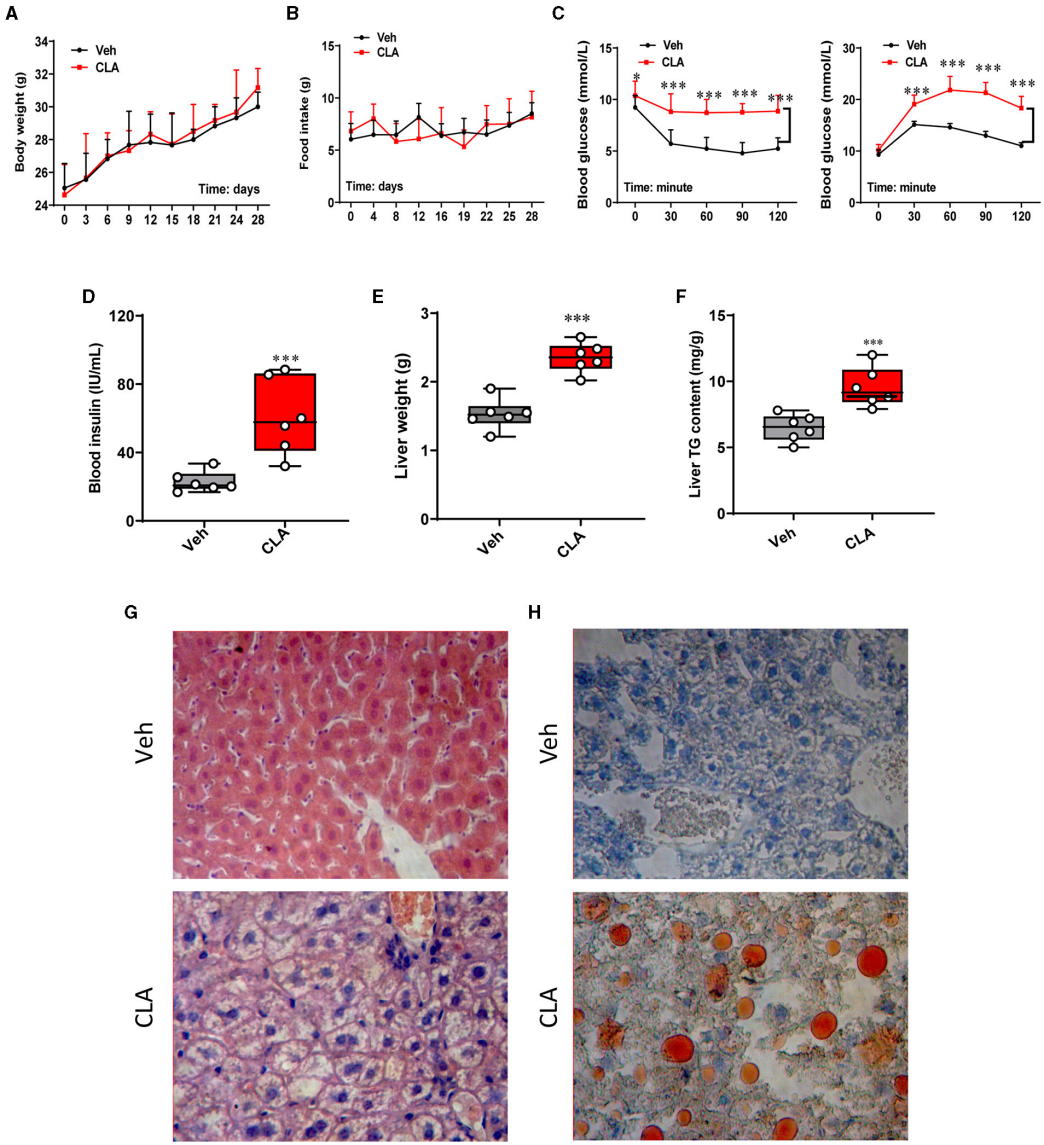
Dietary CLA induces hepatic steatosis in mice. **(A)** Mice were fed either with a control diet/Vehicle or a diet with 1.5% (w/w) CLA supplementation for 28 days, during which the body weight and food intake **(B)** were measured. **(C)** Blood glucose (mmol L-1) following insulin or glucose tolerance test at 0, 30, 60, 90, and 120 min posterior to intraperitoneal injection (*i.p*.) injection of insulin. **(D)** Blood insulin (IU ml-1) levels were measured. **(E)** Liver weight (g) and **(F)** liver total triglycerides content (mg g^−1^). **(G)** Representative images of the liver sections stained with H&E. **(H)** Representative images of the liver sections stained with Oil Red O showing increased lipid deposition in CLA-treated mice. Duplicate slides per animal were analyzed. The data are shown as the means ± SEM, *n* = 6, *p < 0.05, ***p < 0.001 using two-tailed student's *t*-test.

With 1.5% dietary conjugated linoleic acid (CLA) supplementation for 28 days, the changes in animal physiology and lipid metabolism were investigated. This CLA treatment did not affect the body weight or food intake of the mice during the whole period of the experiment compared to the control group (Figures 1A,B). In contrast, the insulin tolerance test/glucose tolerance test **(**ITT/GTT) showed impaired insulin resistance in the CLA-exposed mice compared to the control (Figure 1C), in parallel with the elevated blood insulin levels (Figure 1D). Meanwhile, the CLA treatment dramatically increased the liver weight in mice compared to those of the control group (Figure 1E), along with a significant increase of triglycerides content in the mice fed with CLA (Figure 1F). Histological analysis and Oil Red O staining confirmed the hepatic lipid accumulation in the CLA-treated mice, but not in the control group (Figures 1G,H). These results indicated that dietary CLA supplementation impairs animal body fitness, characterized by a heavier liver, hepatic steatosis, and insulin resistance.

### The Hepatic Circadian Clock Gene Program Is Disrupted in the CLA-Treated Mice

**Figure 2:**
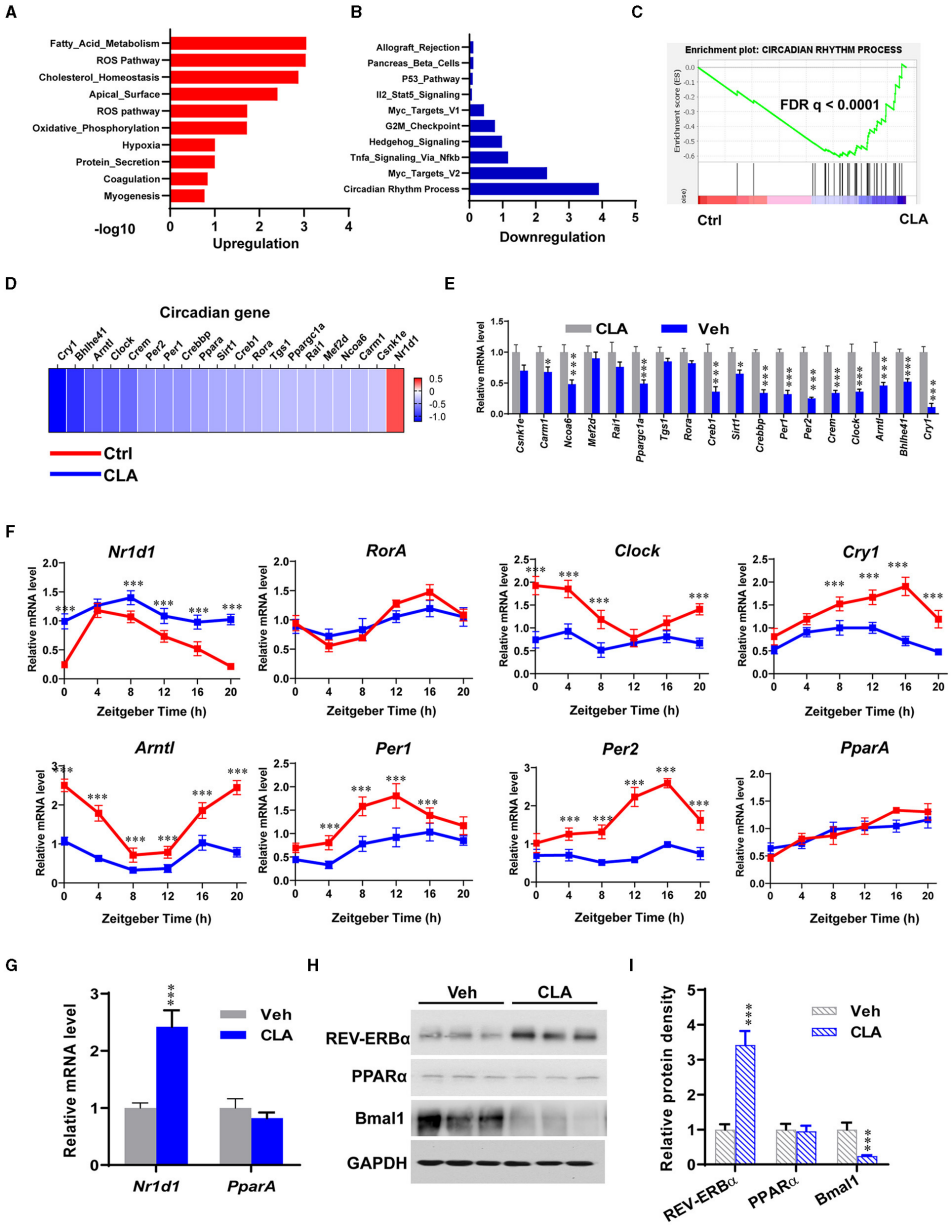
Dietary CLA disrupts the circadian clock gene program in the liver. Gene ontology analysis of the upregulated **(A)** and downregulated **(B)** genes in the livers of CLA mice compared to that of the control groups. Hypergeometric test and Benjamini-Hochberg *p*-value correction was applied. **(C)** GSEA plots depicting the enrichment of genes downregulated in the circadian rhythm process of livers from CLA-treated mice compared to the controls. FDR, false-discovery rate. **(D)** Heatmap of mRNA expression (RNA-seq) changes of the circadian clock in the livers of CLA-treated mice (log2 transformed, normalized to Veh). **(E)** The qRT-PCR analysis confirmed changes of genes involved in the circadian clock in the livers. **(F)** Relative mRNA expression of canonical core clock genes in the livers. Livers were collected at each indicated ZT under a 12-h light/dark cycle. **(G)** Relative mRNA expression of transcription factors *Nr1d1* encoding REV-ERBα and *Ppar*α encoding PPARα, normalized to *Gapdh* expression. **(H,I)** Immunoblotting of REV-ERBα, PPARα, and Bmal1 protein expression normalized to GAPDH levels and quantification of relative protein density. The data are shown as the means ± SEM, *n* = 6 per group, *p < 0.05, ***p < 0.001, using two-tailed student's *t*-test.

Transcriptional profiling of livers was performed to provide the molecular understanding of the CLA-induced nonalcoholic fatty liver disease (NAFLD) in mice. Intriguingly, gene set enrichment analysis (GSEA) revealed that the hallmarks of the circadian clock gene program were strongly downregulated, whereas the fatty acid metabolism pathway was significantly elevated by the CLA treatment compared to the control group (Figures 2A–C). The pathway-focused analysis confirmed that the genes of the circadian program were significantly downregulated in the livers of the CLA-treated mice (Figure 2D). Furthermore, quantitative real-time PCR (qRT-PCR) analysis verified that the key Clock genes including *Per1, Per2, Clock, Arntl*, and *Cry1* were downregulated in the livers of the CLA-treated mice compared to the control group (Figure 2E). To further examine whether the CLA-treatment impairs the liver circadian clock genes rhythmicity, we studied the temporal gene expression in the livers of mice, collected at each indicated zeitgeber time (ZT) (sampled every 4 h) under a 12-h light/dark cycle. The majority of the measured hepatic circadian clock genes lost their rhythmicity in the CLA-treated mice compared to the control, including *NR1D1, Clock, Cry1, Arntl*, and *Per2* (Figure 2F). In contrast, the primary negative transcription factor of the circadian clock NR REV-ERBα (*Nr1d1*) was significantly upregulated at both the transcription and translation levels (Figures 2G–I). In association with the downregulated *Arntl* transcripts, the protein level of Bmal1 was dramatically reduced in the CLA group. We also examined the expression of another NR PPARα, which is a master lipid sensor in the liver. However, there was no difference in the PPARα expression of mRNA or protein between the treatments (Figures 2G–I).

### REV-ERBα and PPARα Drive Circadian Gene Transcription in the Hepatocytes From the CLA-Treated Mice

**Figure 3:**
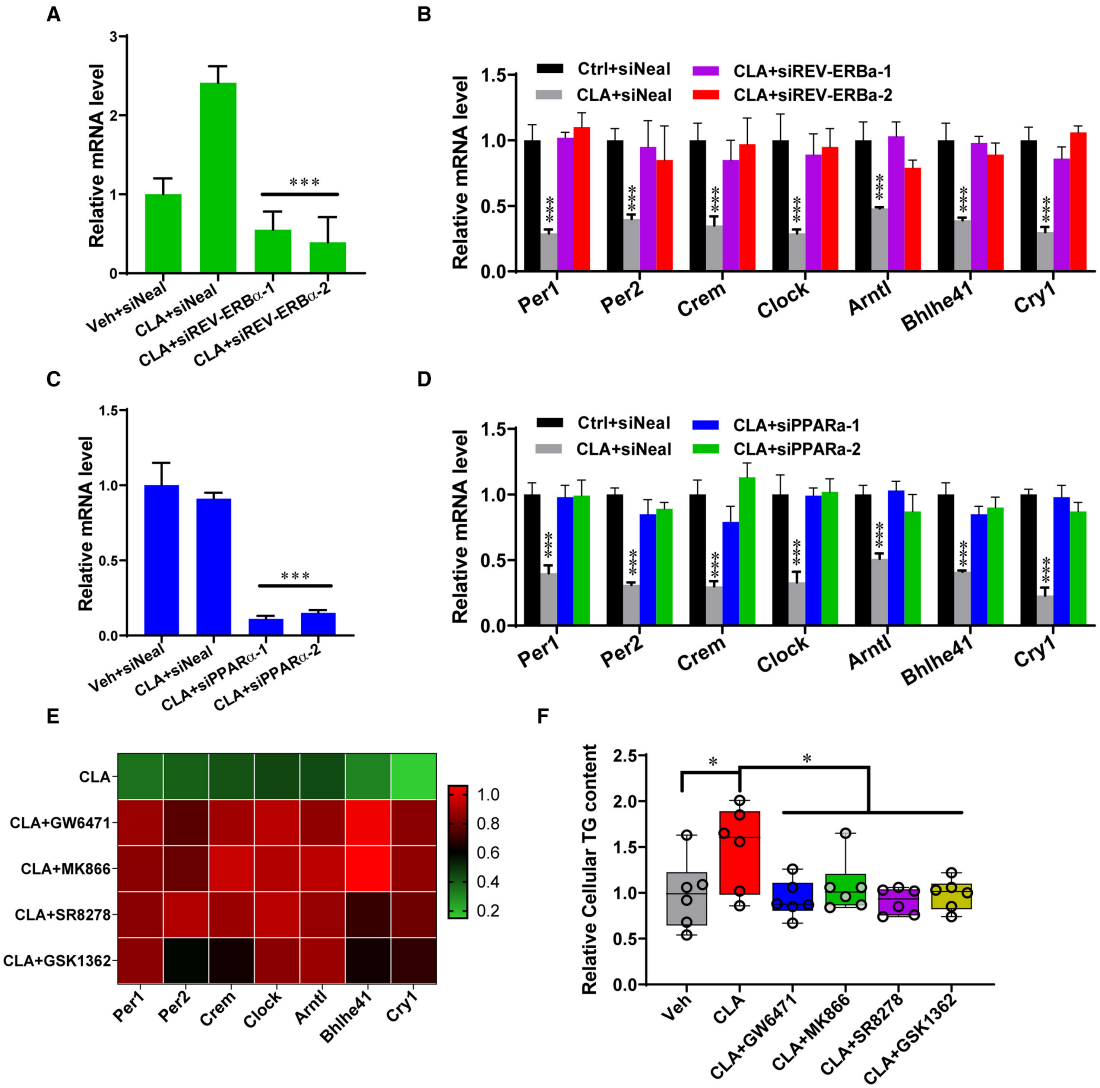
REV-ERBα and PPARα affect circadian genes transcription in the CLA-treated murine hepatocytes. **(A,B)** Relative mRNA levels of REV-ERBα or 7 key genes of the circadian clock **(B)** in Vehicle or CLA-treated mice derived primary hepatocytes treated with siREV-ERBα-1 and siREV-ERBα-2 or control vector. **(C,D)** Relative mRNA levels of PPARα **(C)** or 7 key genes of the circadian clock **(D)** in hepatocytes treated with siPPARα-1 and siPPARα-2 or control vector. **(E)** Heatmaps of fold changes (in log2) of the circadian clock key genes mRNA analyzed by qRT-PCR in murine primary hepatocytes (10 μM of antagonists, GW6471, MK866, SR8278, or GSK1362 treated for 24 h compared to CLA with DMSO). **(F)** Relative total cellular triglyceride contents. The data are shown as the means ± SD, *n* = 6 per group. The experiments were repeated three times. *p < 0.05, ***p < 0.001, using ANOVA with Tukey's *post-hoc* test.

The action of the transcription factors on the circadian gene expression was further investigated. Small interfering RNAs (siRNAs) were used to knockdown REV-ERBα and PPARα in the hepatocytes derived from the CLA-treated mice, respectively (Figure 3). As expected, REV-ERBα knockdown by siREV-ERBα-1/-2 showed that the messenger RNA (mRNA) levels of the key circadian genes *Per1, Per2, Crem, Clock, Arntl, Bhlhe41*, and *Cry1* were restored to that of the control group, compared to the CLA-alone treatment (Figures 3A,B). Interestingly, the downregulated expression of the circadian genes was also switched to the control levels when PPARα was silenced (Figures 3C,D). Similarly, the inhibition of PPARα or REV-ERBα using small-molecule antagonists showed a comparable capacity to increase the key circadian genes expression in the hepatocytes from the CLA-treated mice, as in the siRNA approach (Figure 3E). This was linked to the alteration of lipid output. Thus, the antagonists significantly decreased the CLA-induced high cellular triglycerides levels in hepatocytes (Figure 3F). Together, these results indicate that both REV-ERBα and PPARα have vital functions in driving the circadian gene transcription in response to CLA, even though the endogenous expression of PPARα was unaltered.

### REV-ERBα Recruits Histone Repressors on the Circadian Genes in the CLA-Treated Mice

**Figure 4:**
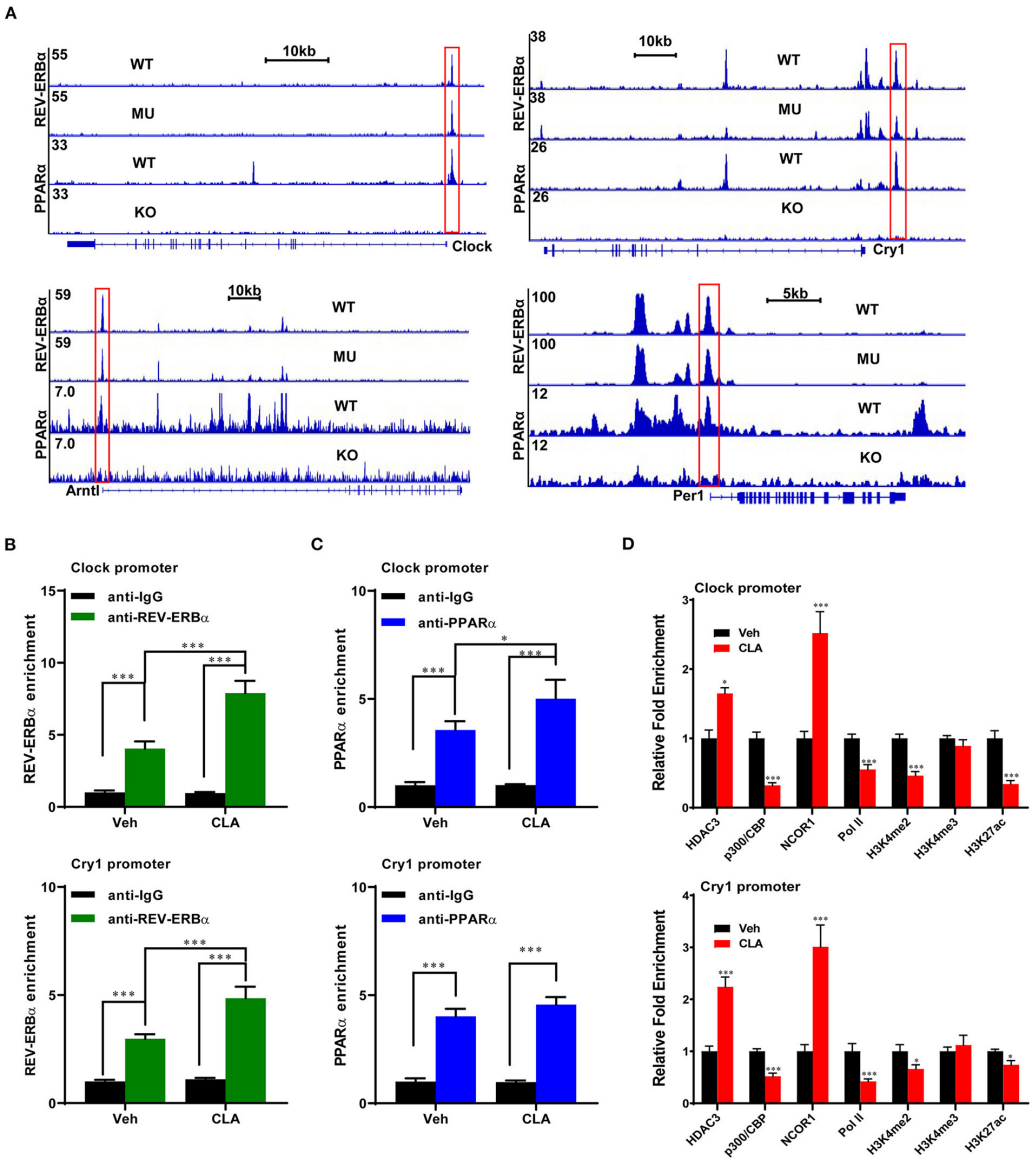
REV-ERBα recruits histone repressors on the circadian genes in the CLA-treated murine hepatocytes. **(A)** Analysis of ChIP-seq dataset in mouse models and ChIP-seq signal visualization of REV-ERBα and PPARα binding at the clock genes, *Clock, Cry1, Per1*. and *Arntl*. **(B,C)** REV-ERBα or PPARα binding to the promoters of *Clock* (upper panel) and *Cry1* (lower panel) determined by ChIP-qPCR in the CLA-treated murine hepatocytes compared to Veh. **(D)** Relative enrichment of cofactors HDAC3, p300, and NCOR1, Pol-II, and histone mark H3K4me2, H3K4me3, and H3K27ac. The data are shown as the means ± SD, *n* = 6 per group. The experiments were repeated three times. *p < 0.05, ***p < 0.001, using the two-tailed student's *t*-test or ANOVA with Tukey's *post hoc* test.

Next, bioinformatic analysis of a previous PPARα and REV-ERBα dataset was performed (34). The key transcription factor, REV-ERBα, was observed to directly bind to the target loci of major circadian genes *Clock, Cry1, Per1*, and *Arntl*. Surprisingly, we found that PPARα also binds to the same loci of the above-mentioned four genes in the mice. The binding enrichments were decreased, when the REV-ERBα DNA binding element was mutated or PPARα was knocked out (Figure 4A). Using the genome-wide analysis and ChIP-qPCR analysis, we confirmed the binding sites of REV-ERBα and PPARα on the gene *Clock* and *Cry1*. The CLA supplementation strongly increased the enrichment of REV-ERBα on both the gene promoters (Figure 4B), and enhanced the PPARα binding on the *Clock* gene (Figure 4C, upper panel) but not on the *Cry1* locus (Figure 4C, lower panel). Subsequently, the repressors of histone deacetylase 3 (HDAC3) and nuclear receptor corepressor 1 (NCOR1) also exhibited higher enrichment at the promoters of *Clock* and *Cry1*, respectively, whereas the occupancy of the coactivator histone acetylase p300 was decreased (Figure 4D). In addition, the transcriptional activation-linked histone marks H3K27ac, H3K4me2, and the RNA polymerase II (Pol-II) recruitment to the target genes were significantly reduced by the CLA treatment. Therefore, our results suggest that CLA enhances the REV-ERBα recruitment and transcriptional repression of the circadian gene program, which results in the inhibition of their transcriptional responses.

### PPARα Dominates REV-ERBα-Mediated Transcription Repression of the Circadian Genes

**Figure 5:**
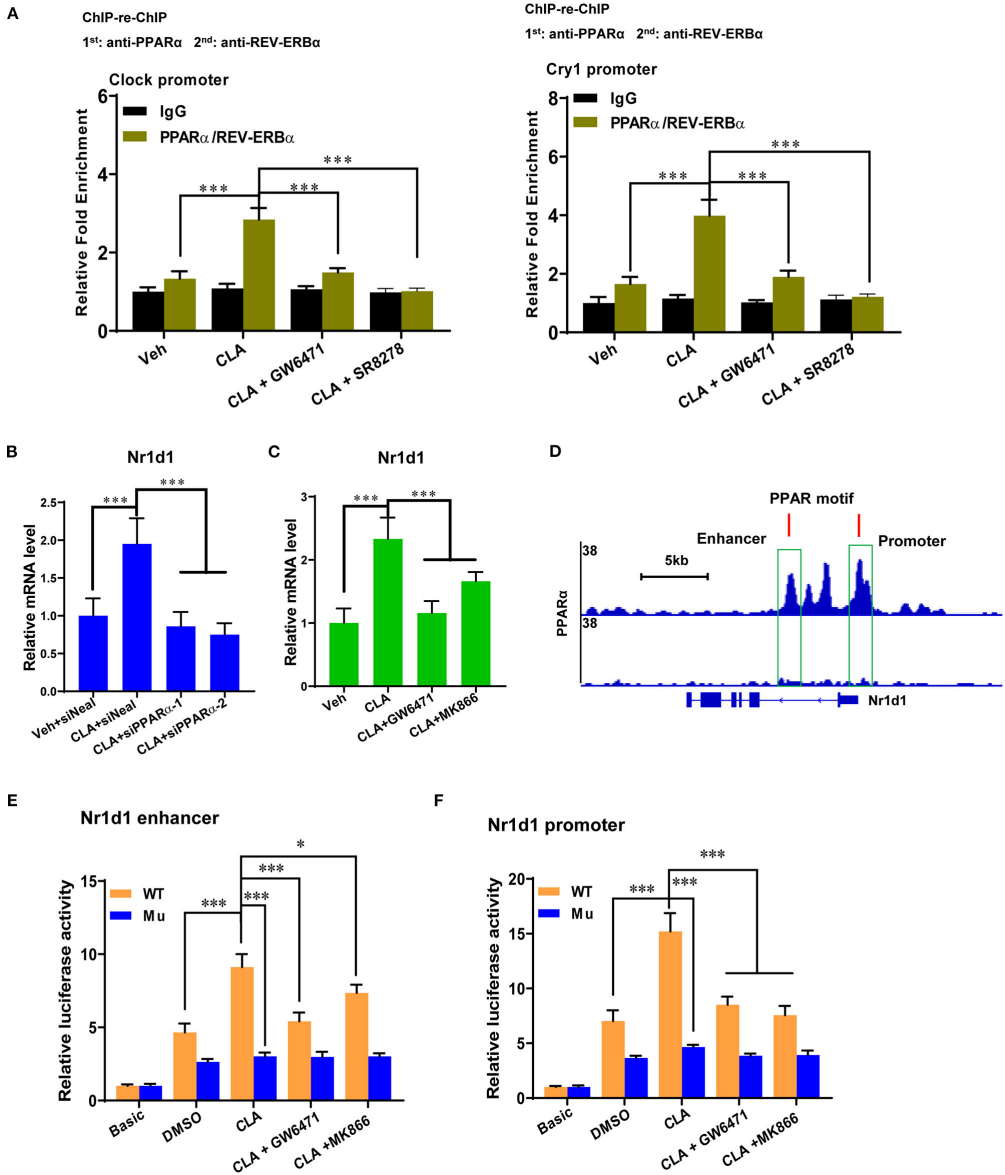
PPARα determines REV-ERBα-mediated transcription repression of circadian genes. **(A)** ChIP-re-ChIP analysis with CLA-treated mice derived primary hepatocytes treated with GW6471, SR8278 or vehicle for 48 h. The cell extracts were used for ChIP and re-ChIP with indicated antibodies. DNA from the second ChIP was analyzed at the promoters of *Clock* and *Cry1* by real-time PCR. The experiments were repeated three times. **(B,C)** Relative mRNA levels of *Nr1d1* (REV-ERBα) in Veh or CLA-treated mice derived primary hepatocytes when PPARα was inhibited by siRNAs or antagonists. **(D)** PPARα-binding motif at the promoter and the enhancer of REV-ERBα gene (*Nr1d1*) in ChIP-seq genome-wide browser analysis. **(E,F)** Relative luciferase expression of PPARα at the *Nr1d1* enhancer or promoter with the wild-type or mutated PPRE in hepatocytes treated with vehicle (DMSO), CLA, cotreatment of GW6471 or MK866 with CLA for 24 h and determined by the reporter gene assay. The data are shown as the means ± SD, *n* = 6. The experiments were repeated three times. *p < 0.05, ***p < 0.001, using two-tailed student's *t*-test or ANOVA with Tukey's *post hoc* test.

To gain a deeper understanding of the interactions between the PPARα and REV-ERBα at the circadian gene promoter, ChIP-re-ChIP was performed in hepatocytes from the CLA-treated mice. The PPARα-chromatin complexes were enriched by the first ChIP with anti-PPARα antibody and were subject to the second ChIP. A strong REV-ERBα association was then detected by the REV-ERBα-specific antibody, but not by the control IgG at the *Clock* and *Cry1* gene promoters, respectively (Figure 5A). Moreover, both the PPARα inhibitor, GW6471 and the REV-ERBα inhibitor, SR8278 were able to reduce the associated enrichments, which indicate that PPARα binds concurrently with REV-ERBα to the circadian genes in the CLA-derived hepatocytes. Importantly, we have provided the proof that PPARα may directly control the REV-ERBα transcription activity by the inhibition of PPARα using the siRNAs approach (Figure 5B), and small-molecule antagonists (Figure 5C). Indeed, the classic PPARα binding motif was found at the promoter and the enhancer of the REV-ERBα gene (*Nr1d1*) in a ChIP-seq genome-wide browser analysis (Figure 5D). To verify the function of this putative PPAR response element (PPRE)-containing site, we performed reporter gene assays with the wild-type or mutated PPRE of *Nr1d1* gene enhancer and promoter. Cell-based reporter assays with constructs including PPRE confirmed the transactivation. The CLA addition was further induced and the antagonists combined with the CLA suppressed the luciferase expression, which was initially higher in the CLA-alone group relative to the basal transactivation (Figures 5E,F). Similar results were observed where the mutation of the Nr1d1-PPRE blocked all responses to CLA and/or antagonists. This suggests that PPARα directly binds to PPRE at the *Nr1d1* gene, thus determining the REV-ERBα-mediated transcriptional activity in response to CLA.

### REV-ERBα Recruits Histone Repressors on the Circadian Genes in the CLA-Treated Mice

**Figure 6:**
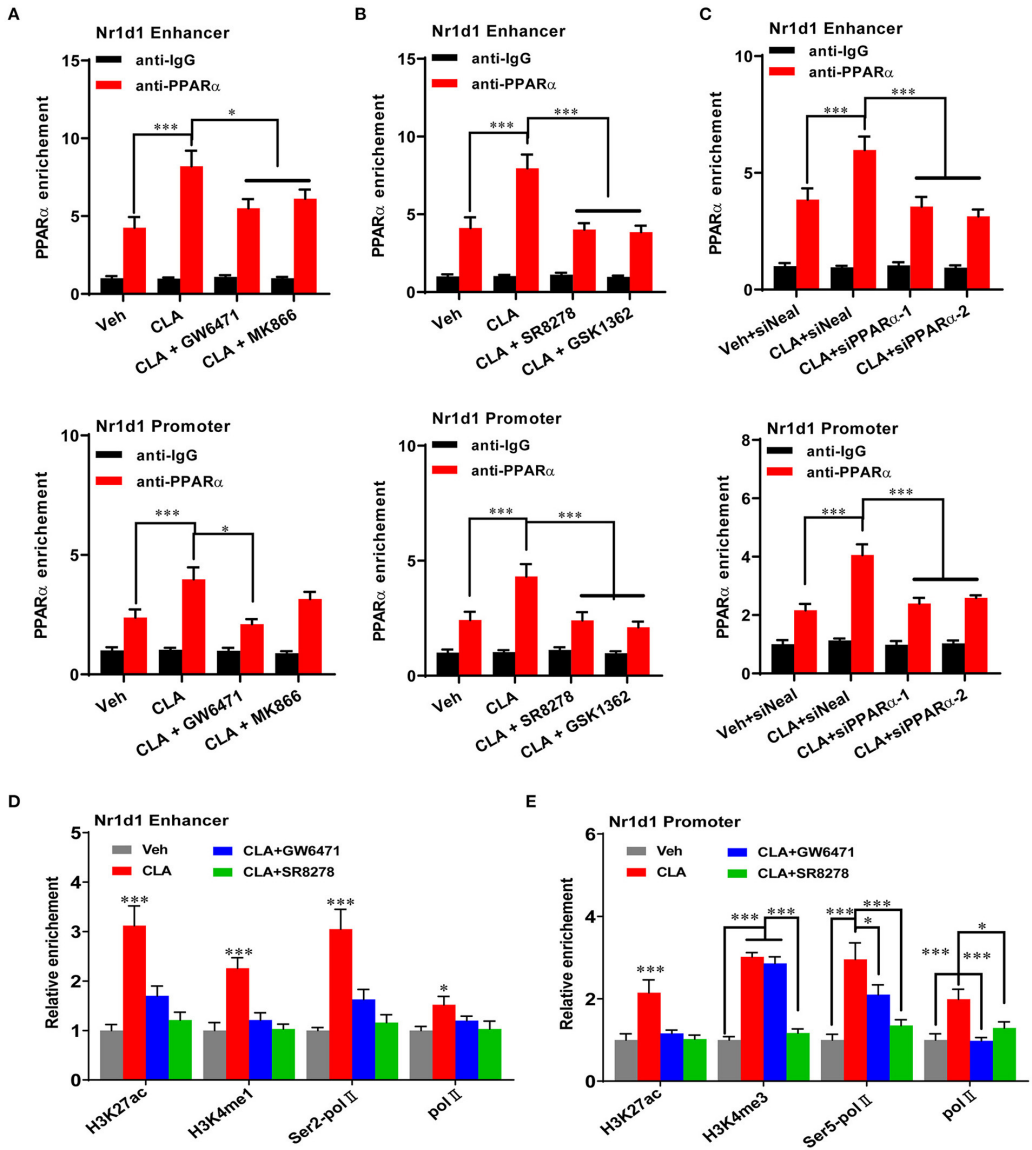
PPARα mediates REV-ERBα transcriptional activation. **(A–C)** PPARα binding to the enhancer of *Nr1d1* (upper panel) and the promoter of *Nr1d1* (lower panel) determined by ChIP-qPCR in the hepatocytes of vehicle control, CLA, or cotreatment of CLA with PPARα antagonists (GW6471, MK866) in **(A)**, or cotreatment of CLA with REV-ERBα antagonists (SR8278, GSK1362) in **(B)**, or CLA with PPARα knockdown in **(C)**. **(D)** Relative enrichment of histone marks H3K27ac and H3K4me1, and Pol-II and Ser2P Pol-II recruitment at the enhancer of *Nr1d1*. **(E)** Relative enrichment of histone marks H3K27ac and H3K4me3, and Pol-II and Ser5P Pol-II recruitment at the promoter of *Nr1d1*. The data are shown as the means ± SD, *n* = 6 per group. The experiments were repeated three times. *p < 0.05, ***p < 0.001, using ANOVA with Tukey's *post-hoc* test.

**Figure 7:**
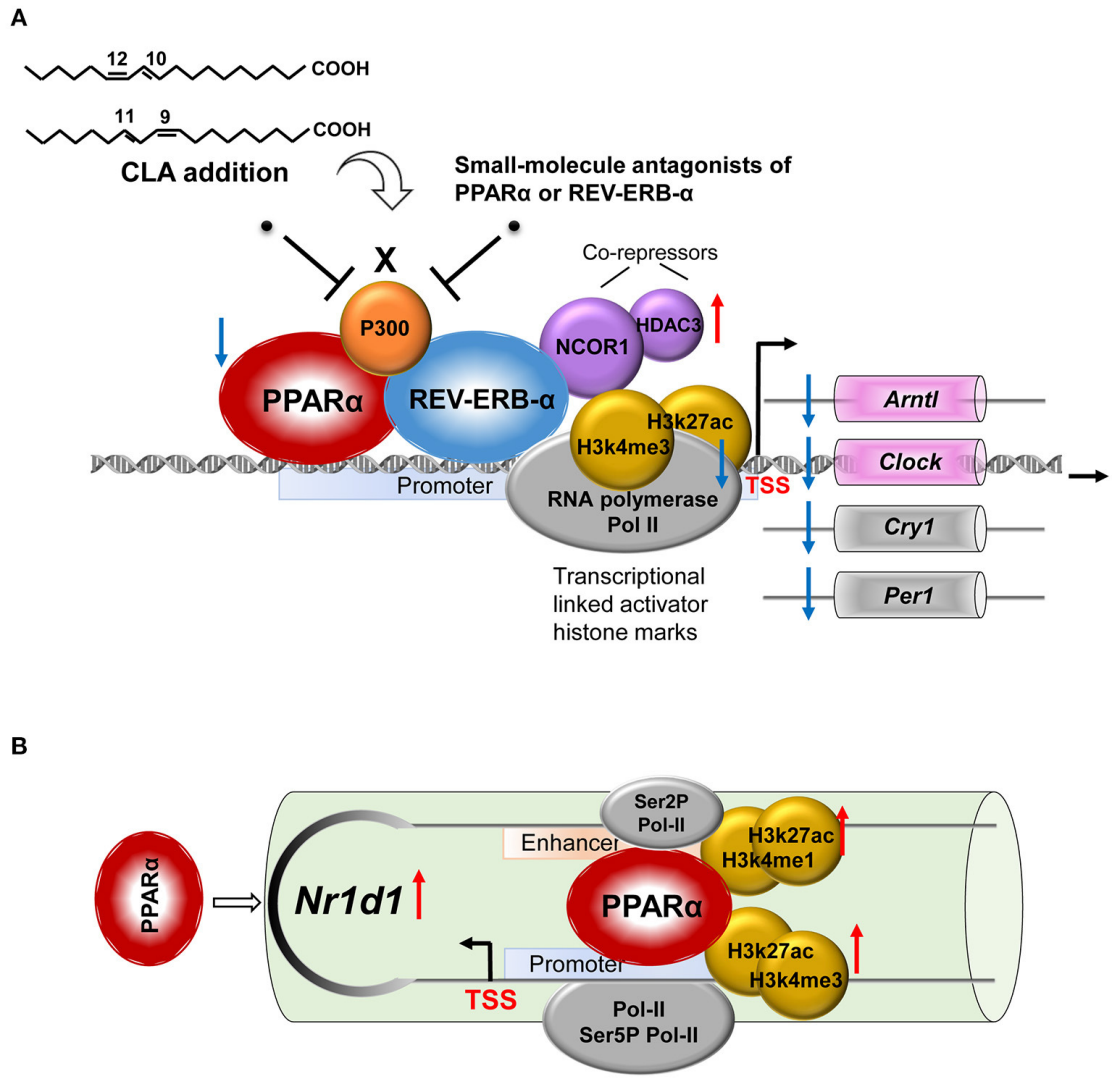
A proposed model for the role of the NR pair, PPARα-REV-ERBα, and its regulation of circadian clock genes in CLA-induced hepatic steatosis. **(A)** CLA-induced activating PPARα and repressive REV-ERBα transcriptional complexes, the coordinate actions of which modulate the circadian clock gene expression. **(B)** The regulation mechanism of PPARα in directing REV-ERBα (*Nr1d1*) expression *via* the recruitment of histone marks and cofactors at both the promoter and the enhancer.

We performed ChIP-qPCR to examine the chromatin modification at the *Nr1d1* gene promoter and enhancer. In association with the gain of *Nr1d1* mRNA occupancy, the CLA treatment enhanced PPARα enrichments both at the Nr1d1 promoter and enhancer (Figure 6). As expected, tool inhibited compounds, such as GW6471 and MK866 against PPARα or SR8278 and GSK1462 against REV-ERBα, exhibited a remarkable efficiency to reduce the binding enrichments at the specific locus (Figures 6A,B). Another evidence that supported the finding is the similar profile we observed when PPARα was inhibited by siRNAs-inducible knockdown (Figure 6C). The histone marks, H3K27ac and H3K4me1 were significantly reduced at the enhancer regions, while H3K27ac and H3K4me3 were reduced at the promoter regions (Figures 6D,E). In addition, the antagonists of PPARα or REV-ERBα also reduced the promoter occupancies of Pol-II, its transcription elongation-associated CTD-ser 2 phosphorylated form (Ser2P Pol-II) and transcription initiation-associated CTD-ser 5 phosphorylated form (Ser5P Pol-II) at the enhancer and the promoter of *Nr1d1*, compared to the CLA alone-treated hepatocytes (Figures 6D,E). Finally, our results showed that the PPARα antagonist quenched the activation of the transcription repressor, REV-ERBα by blocking the PPARα binding to its gene and the associated local histone modification induced by the CLA. Therefore, the results suggest that in CLA-treated hepatocytes, the PPARα plays a dominant role in directing the REV-ERBα expression *via* the recruitment of histone marks and cofactors (Figures 7A,B).

## Discussion

Defining the molecular mechanisms of the circadian clock control may help to better understand how dietary lipids affect animal metabolic health. In this study, we found that the addition of polyunsaturated conjugated linoleic acid (CLA) disrupts the hepatic circadian clock program including the reduced expression of the genes, *Clock* and *Cry1*. It was downregulated by the well-defined negative transcription factor, REV-ERBα, but also requires the PPARα activation and their physical association at the locus of target genes. In particular, we demonstrated that CLA induces the activating PPARα and repressive REV-ERBα transcriptional complexes, simultaneously enhance the corepressors, histone deacetylase 3 (HDAC3) and nuclear receptor corepressor 1 (NCOR1), and reduces the coactivators, p300, RNA polymerase II (Pol II), and the associated local histone modification. During this regulation, the PPARα facilitates the REV-ERBα transcriptional activity by directly binding to PPRE at the *Nr1d1* gene in the CLA-treated hepatocytes. Consequently, we showed that the CLA-induced hepatic triglyceride accumulation can be rescued by small-molecule drugs targeting either PPARα or REV-ERBα with the modified circadian clock program; this may be an alternative strategy to manage the associated metabolic disorders.

It is commonly accepted that excessive amounts of intrahepatic triglyceride accumulation are a prominent feature of nonalcoholic fatty liver disease (NAFLD) (35). In this study, we observed that the dietary CLA supplementation caused enlarged livers with increased triglyceride contents, increased fat depot, and insulin resistance in young mice. In agreement, several studies have demonstrated that feeding mice with CLA results in lipodystrophy, hepatic steatosis, and dyslipidemia (36–38). In contrast, the CLA has been intensively studied for its antiobesity, antidiabetic, and antiatherogenic effects (26, 39). The proposed mechanisms include the CLA-regulation of lipid metabolism, adipogenesis, adipocyte apoptosis, and energy expenditure, whereas the differential effects of CLA are likely to depend on dose (low- or high-duration), (short- or long-term), and animal body phenotype (lean or obese) (26, 40). As CLA supplements that contain isomers cis-9, trans-11, and trans-10, cis-12 are widely used for weight management in humans, it is of importance to improve our knowledge on molecular mechanisms of the CLA action (40) and other polyunsaturated fatty acid (FA). Notably, an earlier study reported that a 6-week CLA (1%) feeding regimen disrupts the normal day-night rhythm of respiratory quotients in mice and results in increased FA oxidation at night and impairment of white adipose tissue (WAT) metabolism (41). We discovered that dietary CLA supplementation for 28 days already caused alterations of the circadian rhythm process, including the key clock genes, *Per1, Per2, Crem, Clock, Arntl, Bhlhe41*, and *Cry1* in the liver of mice with the onset of steatosis, while high-fat diet has been shown to induce metabolic abnormalities involving the NR RORα and REV-ERBα, and affects the expression of *Clock* and *Bmals* during 12 weeks of experiment (42).

The maintenance of a proper circadian clock program is important that perturbations of the circadian rhythm predispose individuals to metabolic disorders, cardiovascular diseases, and tumor development (43, 44). In turn, changes in physiological processes, such as food variations including CLA supplementation in this study could reprogram the circadian clock, likely through transcriptional mechanisms. As CLA-induced elevation of the REV-ERBα expression resulted in reduced clock gene messenger RNA (mRNA) expression, this confirmed the role of REV-ERBα as a transcriptional repressor in the liver (45). We further demonstrated that multiple components are involved in the molecular clock modulation by CLA. On top of the increased binding of REV-ERBα on the *Clock* and *Cry1* genes, the CLA enhanced the recruitment of histone repressors, NCOR1, and HDAC3 at the binding sites. Alternatively, the transcriptional activation-linked histone marks H3K27ac and H3K4me2, and coactivators, p300 and Pol II were reduced by CLA, suggesting a two-armed regulation apparatus. Although like other nuclear receptor (NR), REV-ERBα is activated by ligands, such as naturally occurring heme (16), it lacks the canonical NR-activation domain (46). It is unclear whether ingested CLA can directly regulate REV-ERBα expression in the liver. Studies of REV-ERB deletion in mice (REV-ERBα and/or REV-ERBβ) have shown a mild rhythmic phenotype and partially impaired metabolisms in various tissues (13, 14, 47). Using genetic and pharmacological approaches to inhibit REV-ERBα expression in hepatocytes, we revealed an altered clock gene program and reduced triglyceride accumulation in a CLA-specific context. We and other researchers thereby suggest that REV-ERBα is an essential component of the clock machinery, but maybe partially redundant (13, 48). Notably, overexpression of REV-ERBα in the liver of the mouse leads to the suppression of 90% of cycling transcripts, implying a broader impact on circadian rhythm than deletion (49). Therefore, additional or complementary roles of REV-ERBα and its paralog, REV-ERBβ needs to be addressed.

Dietary CLA when ingested is readily assimilated into multiple cell types, which directly influences their metabolism. For 6 weeks of CLA administration in young mice, ~1% of CLA isomer has been shown to be incorporated into the heart, the liver, and the peripheral tissues (50). We hypothesized that in this study, CLA modulates the circadian gene program, in part, by a mechanism depending on the activation of PPARα. This nuclear receptor (NR) is well-known as the master lipid sensor (51, 52), and several isomers of CLA are known as high-affinity ligands and activators of PPARα (53, 54). By using small-molecule antagonists of PPARα and siPPARα-1/-2, we provide evidence that PPARα participates in the REV-ERBα recruitment of histone repressors on the circadian genes and associated triglyceride production increment in response to the CLA. In addition to cooccupying the binding region with REV-ERBα on the clock genes, the PPARα controls the transcriptional activity of REV-ERBα in the CLA-hepatocytes by binding to the *Nr1d1* gene at PPAR response element (PPRE), at both the promoter and the enhancer. We thereby propose a unique “NR crosstalk” of PPARα-REV-ERBα in the regulation of the circadian clock gene program in the CLA-induced hepatic steatosis. Both REV-ERBα and PPARα could function as sensors for the metabolic state of the cell and may entrain the clock to metabolic cues (45). Under specific nutritional challenges, non-cyclic genes including *PPAR*γ are reported to become circadian through the cyclic activation of surrogate transcription pathways, which underline the plasticity of the clock system (28). We suggest that under CLA treatment, the NR pair recruited coregulator complexes to specific regions of the *Clock* and *Cry1* genes, can, in turn, impact the epigenome, that is, the nuclear corepressor 1 (NCOR1) may interact with NR proteins, as well as HDAC3, which deacetylates histone protein tails to create a repressive chromatin environment (8). This is supported by the chromatin immunoprecipitation sequencing (ChIP-seq) analysis of mouse liver that revealed a significant overlap of HDAC3, NCOR, and REV-ERBα on target genes, which is inversely related to histone acetylation and Pol II recruitment (5, 8).

The process of circadian rhythm draws increasing attention due to the growing epidemic of NAFLD and associated metabolic disorders. In this study, we revealed that the NR REV-ERBα serves as a key link between the dietary CLA-induced hepatic steatosis and the downregulation of the circadian gene program in the liver, while another NR PPARα serves as the major CLA respondent and facilitates the REV-ERBα-mediated transcription repression of the circadian genes and its recruitment of histone repressors in the CLA-treated mice. Our results suggest that both the REV-ERB antagonist and the PPARα antagonist may be used as therapeutics to reset diet-associated disruption of the circadian rhythm and reestablish the metabolic balance.

## Data Availability Statement

All data supporting the findings of this study are available within the article, in Supplementary Information, or from the corresponding author upon request. All RNA-seq data generated in this study are deposited in the Gene Expression Omnibus (GEO) database under the accession numbers “GSE159219” (https://www.ncbi.nlm.nih.gov/geo/query/acc.cgi?acc=GSE159219).

## Ethics Statement

All experiments involving animals were reviewed and approved by the Animal Care and Use Committee of Yangzhou University (YZUDWSY 2017-09-06).

## Author Contributions

H-YL and DC: conceptualization. DC, HG, YL, YY, KL, HL, KZ, and BZ: methodology. DC, HG, YL, KZ, HL, YY, BZ, PH, HW, and WB: investigation. H-YL and DC: writing original draft. H-YL, PH, HW, WB, and DC: review and editing. DC: funding acquisition. H-YL, HG, BZ, KZ, HW, and DC: resources. DC: supervision. All authors contributed to the article and approved the submitted version.

## Funding

This study was supported by the National Natural Science Foundation of China (32002243), the Natural Science Foundation of Jiangsu Province (BK20200932), the Natural Science Foundation of the Higher Education Institutions of Jiangsu Province (20KJB230001), the Breeding and Reproduction Innovation Team of Jiangsu Province Modern Agriculture (Swine) Industry Technical System (JATS [2020] 4400), and the Priority Academic Program Development of Jiangsu Higher Education Institutions (PAPD).

## Conflict of Interest

HW is employed by the Baijiu Science and Research Center, Sichuan Swellfun Co., Ltd., China. The remaining authors declare that the research was conducted in the absence of any commercial or financial relationships that could be construed as a potential conflict of interest.

## Publisher's Note

All claims expressed in this article are solely those of the authors and do not necessarily represent those of their affiliated organizations, or those of the publisher, the editors and the reviewers. Any product that may be evaluated in this article, or claim that may be made by its manufacturer, is not guaranteed or endorsed by the publisher.

## Abbreviations

ChIP: chromatin immunoprecipitation
CT: circadian time
CLA: conjugated linoleic acid
CRY: cryptochrome
DKOs: double-knockout mice
FA: fatty acid
GSEA: gene set enrichment analysis
GTT: glucose tolerance test
H&E: hematoxylin and eosin
HDAC3: histone deacetylase 3
*i*. *p*.: intraperitoneal
ITT: insulin tolerance test
mRNA: messenger RNA
NAFLD: nonalcoholic fatty liver disease
NRs: nuclear receptors
NCOR1: nuclear receptor corepressor 1
PER: PERIOD
RNA: polymerase II (Pol-II)
siRNAs: Small interfering RNAs
TTFL: transcription-translation feedback loop
WAT: white adipose tissue
ZT: zeitgeber time.
