# Supplementary material for: Dietary Conjugated Linoleic Acid Modulates the Hepatic Circadian Clock Program via PPARα/REV-ERBα-Mediated Chromatin Modification in Mice
Source: Front Nutr. 2021 Oct 15;8:711398. doi: 10.3389/fnut.2021.711398 (PMC8553932; doi:10.3389/fnut.2021.711398)
Supplement: Supplementary file 1 [file Table_1.DOCX]

| Primer | Sequence（5'-3'） |
| --- | --- |
| *Per2*-F | CTGCTTGTTCCAGGCTGTGGAT |
| *Per2*-R | CTTCTTGTGGATGGCGAGCATC |
| *Arntl*-F | ACCTCGCAGAATGTCACAGGCA |
| *Arntl*-R | CTGAACCATCGACTTCGTAGCG |
| *Nr1d1*-F | CAGGCTTCCGTGACCTTTCTCA |
| *Nr1d1*-R | TAGGTTGTGCGGCTCAGGAACA |
| *Per1*-F | GAAACCTCTGGCTGTTCCTACC |
| *Per1*-R | AGGCTGAAGAGGCAGTGTAGGA |
| *Per3*-F | CACAGACATCGAAGGAGGTGCT |
| *Per3*-R | CTTACACGCCACGGCAACACTT |
| *Cry1*-F | GGTTGCCTGTTTCCTGACTCGT |
| *Cry1*-R | GACAGCCACATCCAACTTCCAG |
| *Ghrl-*F | GAAGCCACCAGCTAAACTGCAG |
| *Ghrl*-R | CTGACAGCTTGATGCCAACATCG |
| *Clock*-F | GGCTGAAAGACGGCGAGAACTT |
| *Clock*-R | GTGCTTCCTTGAGACTCACTGTG |
| *Suv39h2*-F | CCATAAACGCTGGAGAAGAGCTG |
| *Suv39h2*-R | CTGCAAGTCTCGGCTCCACATT |
| *Clock*-promoter-F | GAATGTGAGCGAGGGAGGAGT |
| *Clock*-promoter-R | GCTTTCCCTGGGTCAAATGT |
| *Cry1*-promoter-F | AACTTCCTTATGCCACTTCC |
| Cry1-promoter-R | GCTGTCCTAGCACAGACTAG |
| *Nr1d1*-promoter-F | GCCCACCTTGTCACCATAGCAA |
| *Nr1d1*-promoter-R | GCAAGCTTTACTGCTCCGTG |
| *Nr1d1*-enhancer-F | GCAATACACATCTAGATGGAGAGC |
| *Nr1d1*-enhancer-R | GCAGGGCAACCTTAAAATAGC |

# SupplementaryTables

Supplementary Table 1 Primers used in this study
